# Prolonged Severe CD4+ Lymphocytopenia and Hypogammaglobulinemia in Patients With Evans’ Syndrome: A Case Report

**DOI:** 10.7759/cureus.75283

**Published:** 2024-12-07

**Authors:** Takashi Kurokawa, Naoto Imoto, Hideki Muramatsu, Kurahashi Shingo

**Affiliations:** 1 Department of Hematology, Okazaki Municipal Hospital, Okazaki, JPN; 2 Department of Hematology and Oncology, Toyohashi Municipal Hospital, Toyohashi, JPN; 3 Department of Pediatrics, Nagoya University Graduate School of Medicine, Nagoya, JPN

**Keywords:** autoimmune hemolytic anemia (aiha), cd4+ lymphocytopenia, common variable immunodeficiency (cvid), evans’ syndrome, hypogammaglobulinemia, late-onset combined immune deficiency

## Abstract

Primary immunodeficiency (PID) is one of the causes of secondary autoimmune hemolytic anemia (AIHA) and Evans’ syndrome (ES). Serum immunoglobulins should be tested in patients with AIHA/ES, as common variable immunodeficiency is the most common PID of secondary AIHA/ES. However, it is not fully understood how immunodeficiency is assessed, in addition to serum immunoglobulins. Here, we present the case of a 34-year-old man with prolonged severe CD4^+^ lymphocytopenia and hypogammaglobulinemia in patients with ES despite repeated negative tests for human immunodeficiency virus antibodies. His CD4^+^ cell count remained below 60/µL for 56 months after treatment completion, including steroid and rituximab therapy. A gene panel test for immunodeficiency using next-generation sequencing did not reveal any pathogenic gene variants. He has been using continuously trimethoprim-sulfamethoxazole to prevent pneumocystis pneumonia due to severe CD4^+^ deficiency. This case highlights the need for a CD4^+^ cell count in some patients with AIHA/ES, such as those with hypogammaglobulinemia or recurrent infections.

## Introduction

Evans' syndrome (ES) is a rare autoimmune disorder where the immune system mistakenly attacks red blood cells and platelets, leading to concurrent or sequential autoimmune hemolytic anemia (AIHA) in which red blood cells are destroyed by the body's own antibodies and immune thrombocytopenia (ITP)which causes low platelet counts due to immune system attacks on platelets. ES accounts for 0.3-7% of AIHA cases and approximately 2-2.7% of ITP cases [1-3]. Approximately 20-50% of AIHA and ES are secondary cases such as immunological or lymphoproliferative disorders [1-3].

It is essential to distinguish between primary and secondary AIHA/ES as secondary AIHA/ES requires specific therapy for each disease, affecting treatment strategies and patient outcomes [4]. In addition, AIHA/ES is frequently challenged by infectious complications, mainly because of immunosuppressive treatment and background disease [3,5]. One observational multicenter study showed that 33% of patients with ES experienced infectious episodes, mainly grade 3 or 4 (77%), with fatal events [3]. To prevent and treat these infections, it is necessary to know exactly what underlying diseases are present and what the risk of infection is.

Primary immunodeficiency (PID), a group of disorders in which parts of the immune system are missing or malfunctioning, is one of the causes of secondary AIHA/ES [1-3]. In the immune system, B cells, which produce antibodies, and T cells, which directly attack infected cells and help coordinate immune responses, are the components that provide antigen-specific acquired immunity. The abnormality of the immune system can cause recurrent infections, serious illnesses, and opportunistic infections. It also causes autoimmune reactions and excessive immune responses due to immune dysregulation [6].

PID is mostly diagnosed in children but also occurs in adults. Common variable immunodeficiency (CVID), characterized by low levels of antibodies and increased susceptibility to infections, is the most common PID complicated by AIHA/ES [1-3]. Thus, checking immunoglobulin class quantification in the serum before intravenous immunoglobulin (IVIg) has become standard clinical practice [1-4]. Furthermore, CVID is not the only PID that can cause AIHA/ES; other regulatory disorders can also cause ES. For example, autoimmune lymphoproliferative syndromes (ALPS) are known to be associated with AIHA/ES as a characteristic clinical feature [7,8]. Good syndrome and severe immunoglobulin A (IgA) deficiency are also known to cause AIHA in adults [9,10]. However, PIDs other than CVID that cause secondary AIHA/ES are rare in adults and can easily be overlooked due to lack of adequate recognition.

It has been reported that 8.9% of CIVD is presented with late-onset combined immune deficiency (LOCID), defined as a form of PID that occurs in adults and involves both antibody and T-cell deficiencies [11]. However, measures of T-cell deficiency such as CD4+ counts are not routinely measured outside of specific contexts such as human immunodeficiency virus (HIV) infection or transplant therapy. CD4+ T cells are crucial for defending against infections, and their deficiency can lead to severe immunodeficiency. Therefore, checking the CD4 count can be crucial in detecting conditions such as LOCID in patients with AIHA/ES.

Our case demonstrated severe CD4 lymphocytopenia with hypogammaglobulinemia throughout the course of the disease, even after treatment completion. It indicates the usefulness of measuring CD4 counts in specific patients with ES/AIHA and hypogammaglobulinemia.

## Case presentation

A 34-year-old man presented to our clinic with dizziness and malaise. He had a history of symptomatic epilepsy and head injury and had been taking phenytoin for more than 10 years. No other relevant past or family history such as opportunistic infection, autoimmune diseases, or unexplained illnesses was reported. His conjunctiva was pale. The lymph nodes and liver were not palpable on the body surface, but the spleen was palpable with a single transverse finger. There were no abnormalities of the oral mucosa or skin. Blood tests revealed macrocytic anemia (hemoglobin, 4.0 g/dL), lymphocytopenia (absolute lymphocyte count, 436/μL), and thrombocytopenia (platelet count, 48,000/μL). Additional tests showed high lactate dehydrogenase, low haptoglobin levels, high indirect bilirubin levels, and positive antiglobulin test results. The results also showed decreased immunoglobulin (IgG) (412 mg/dl) and IgA (26 mg/dl) levels (Table 1).

**Table 1:** Blood test before treatment

Variable	Patient values	Reference range
White blood cell count (/µL)	6,230	3300-8600
Neutrophil (%)	83	38.5-80.5
Lymphocyte (%)	7	16.5-49.5
Red blood cell count (million/µL)	1.07	4.4-5.6
Hemoglobin (g/dL)	4	13.7-16.8
Hematocrit (%)	12.2	40.7-50.1
Mean corpuscular volume (fL)	114	83.6-98.2
Reticulocyte count (%)	20.9	0.5-1.5
Platelet count (1,000/µL)	48	158-348
Lactate dehydrogenase (U/L)	552	124-222
Total bilirubin (mg/dL)	2.9	0.4-1.5
Direct bilirubin (mg/dL)	0.9	0.0-0.5
Immunoglobulin (IgG) (mg/dl)	412	861-1747
Immunoglobulin (IgA) (mg/dl)	26	93-393
Haptoglobin (mg/dl)	<5.0	19-170
Direct antiglobulin test IgG	4+	(-)
Direct antiglobulin test C3b,C3d	3+	(-)

We did not check pre-treatment CD4+ and CD8+ T-cell counts as we did not know the importance of assessing immunodeficiency status other than immunoglobulin in patients with AIHA/ES. 

There was no organ failure, and no schistocytes were found in the blood smear. A small nodule and surrounding interstitial ground glass opacity in the S6 region of the lower lobe of the left lung were observed on computed tomography (CT). There was splenomegaly with a maximum diameter of 11.7 cm and no other abnormalities on CT. Bone marrow examination showed hypercellularity with erythroblastic hyperplasia (erythroid, 63.2%). There were no malignant cells, dysplasia, or increase in blasts. Cytogenetic analysis showed no abnormalities: 46, XY (20/20). This result excluded aplastic anemia and myelodysplastic syndrome. Unfortunately, the flow cytometry test was not included in this study.

The patient was diagnosed with warm AIHA and ITP, which was defined as ES. Hypogammaglobulinemia indicates the presence of CVID as a background disease. Tests for HIV antibodies were negative, and no past or family history of immunodeficiency was noted.

The patient was started on prednisone (PSL) 1 mg/kg on day 1, but his anemia did not improve. Steroid pulse therapy (methylprednisolone, 1000 mg/day for three days) was initiated on day 4. On day 8, his CD4+ (15/µL) and CD8+ cell counts (87/µL) were found to be severely decreased, which was believed to be attributable to steroid treatment, although they had not been measured before treatment. On day 8, we examined the peripheral flow cytometry and found that 94.8% were CD20-positive B cells in the lymphocyte area. High-dose gamma-globulin therapy (20 g for five days) was administered on day 13. Rituximab (375 mg/m^2^ weekly × four doses) was administered on day 19 to maintain the treatment effect. As a result of treatment, hemoglobin levels became normal and platelets increased to over 100,000/µL approximately one month after initial diagnosis. After discharge, phenytoin was stopped and levetiracetam was started because of suspected drug-induced hypogammaglobulinemia.

After the recovery of red blood cell and platelet counts, the dose of PSL was gradually reduced from day 30. Four months after the diagnosis, the patient’s CD4+ (14/µL) and CD8+ (135/µL) cell counts remained low. A re-examination of the HIV antibody test results was negative. The patient underwent an immunological examination. Lymphocyte stimulation tests with phytohemagglutinin and concanavalin A showed a decrease in the stimulation index to 21.4 (reference range >147.5) and 23.7 (reference range >38.1), respectively, indicating cellular immunodeficiency. He had been vaccinated against measles as a child and his measles antibody titer was 4.9 (reference range <4.0). A gene panel test for immunodeficiency using next-generation sequencing (NGS) [12] did not identify any pathogenic gene variants.

**Figure 1:**
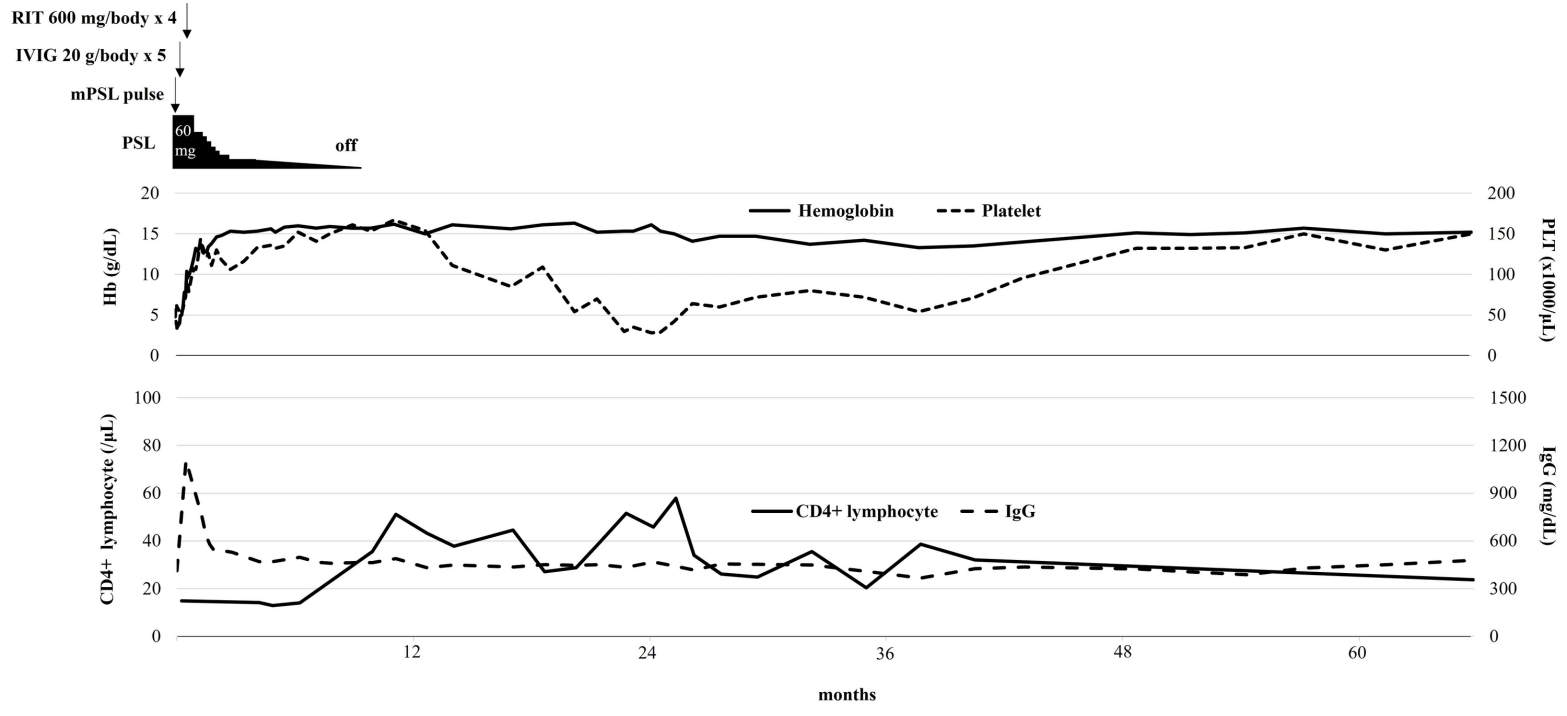
Clinical course Hb, hemoglobin; IgG, immunoglobulin G; IVIG, intravenous immunoglobulin; mPSL, methylprednisolone; PLT, platelet; PSL, prednisolone; RIT, rituximab

Ten months after the diagnosis, steroids were withdrawn, and the CD4+ and CD8+ cell counts were 36 /µL and 372 /µL, respectively. Thereafter, ES did not recur, and the CD4+ cell count remained below 60/µL for 56 months after the end of treatment (Figure 1). 

**Figure 2:**
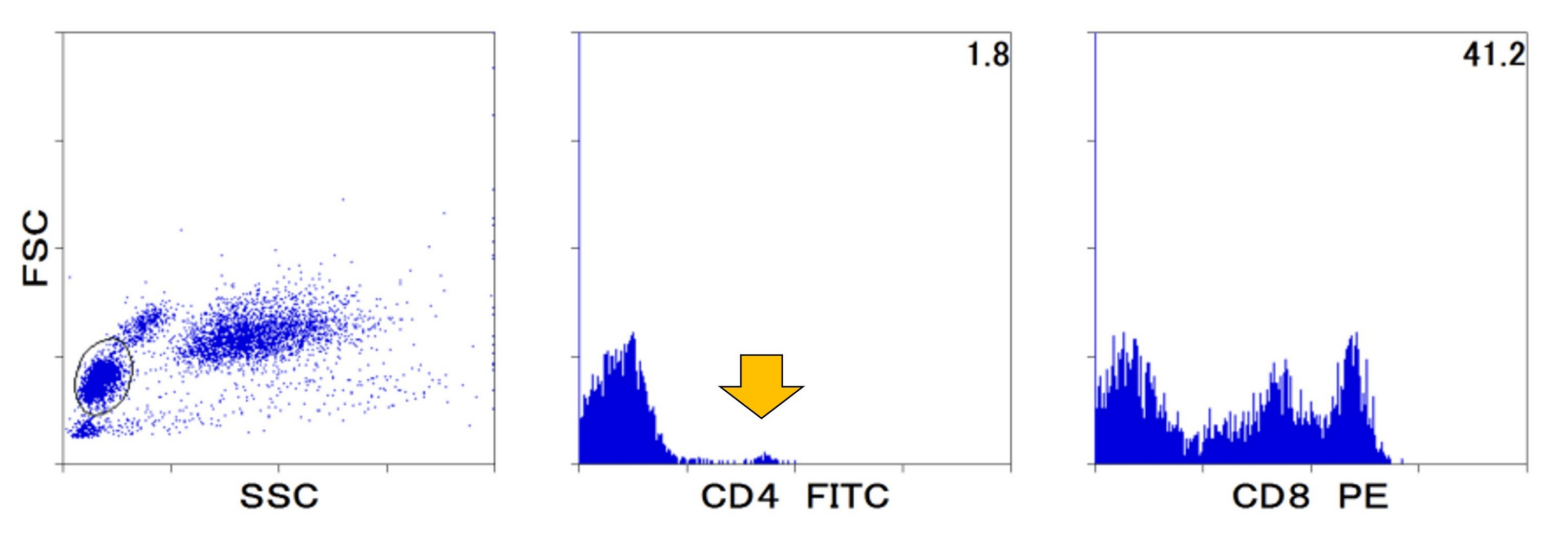
Flow cytometry results for CD4+ and CD8+ counts 56 months after the completion of treatment The CD4+/CD8+ ratio was 0.04 (reference range, 0.4-2.3) FITC, fluorescein isothiocyanate; FSC, forward scatter; PE, phycoerythrin; SSC, side scatter

At the final examination at 56 months after the end of steroids, lymphocyte stimulation tests with phytohemagglutinin and concanavalin A showed a decrease in the stimulation index to 4.0 (reference range >147.5) and 8.1 (reference range >38.1), indicating continued severe cellular immunodeficiency. The results of CD4+ and CD8+ cell analyses at that time are shown in Figure 2.

There were no documented infectious events, no hospitalizations, and no antibiotics administered during the treatment and follow-up from the date of the first visit. No treatment-related adverse events were observed. CT was reviewed at a recent outpatient visit and showed no splenomegaly, no inflammatory or nodular findings in the lungs, and no lymphadenopathy or other infectious lesions.

**Figure 3:**
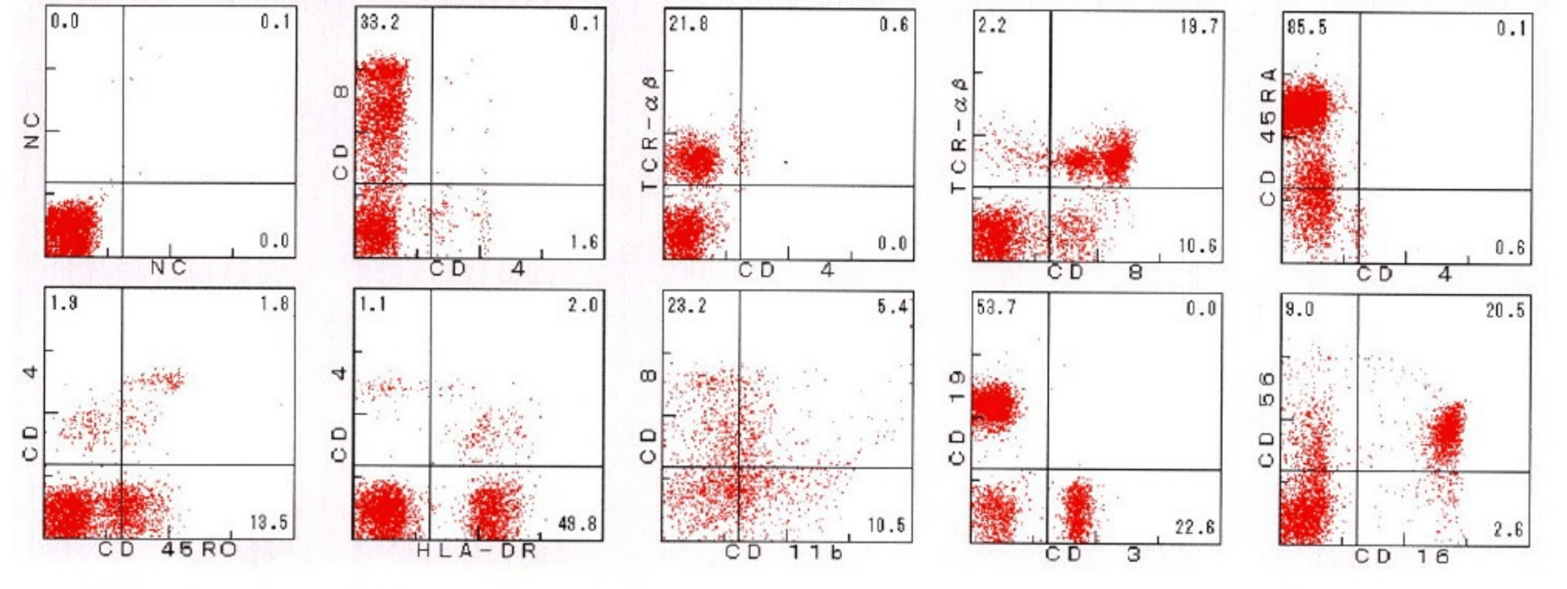
Flow cytometry of peripheral blood at 56 months after end of the treatment

Figure 3 shows the result of flow cytometry of peripheral blood used to assess immunodeficiency status. In lymphocyte area, there were fewer CD4+ cells than CD8+ cells. Although no system directly measures CD3+ TCRαβ+CD4-CD8- cells due to insurance issues, the ratio of CD4+ cells to TCRαβ+CD8- cells is almost identical, suggesting no increase in CD3+ TCRαβ+CD4-CD8- cells. In CD4+ cells, small numbers of CD45RA+ naive T cells (0.1%), CD45RO+ memory T cells (1.8%), and HLA-DR+ activated T cells (2.0%) were observed, while in CD8+ cells, CD11b+ suppressor T cells (5.4%) and CD11b+ activated T cells (23.2%) were observed. CD19+ B cells were observed in 53.7% and CD56+CD16+ NK cells in 20.5%.

The patient was diagnosed with LOCID [6,8] and has been attending the hospital taking only trimethoprim-sulfamethoxazole to prevent pneumocystis pneumonia (PCP). AIHA/ES has not progressed from the initial diagnosis to date.

## Discussion

This case showed prolonged severe CD4+ lymphocytopenia and hypogammaglobulinemia in a patient with ES, even after treatment completion. There are several differential diagnoses for PID causing ES. CVID is the most common disease of PID causing ES, but the patient did not meet the diagnostic criteria for CVID due to evidence of profound T-cell deficiency [13]. Idiopathic CD4 depletion can also be excluded because unlike in this patient, immunoglobulin levels are typically normal or slightly depressed [14,15]. We can rule out Good syndrome because no thymus gland was identified on the pre-treatment CT [9]. ALPS syndrome can also be excluded because there was no persistent lymphadenopathy or splenomegaly, lymphocytopenia was present at initial diagnosis, and there were no apparent CD3+ TCRαβ+ CD4- CD8- double negative T cells on flow cytometry [7]. As a cause other than PID, we should consider drug-induced ES and hypogammaglobulinemia. There are several reports that phenytoin can cause hypogammaglobulinemia [16,17]. However, we believe that the likelihood of drug-induced events is low because the severe T-cell deficiency and hypogammaglobulinemia persisted after switching phenytoin to levetiracetam. Overall, this case can be categorized as LOCID as a distinct entity of CVID [11].

A French study group reported that 28 out of 313 patients (8.9%) with CVID were categorized as having LOCID due to severe defects in cell-mediated immunity or had a CD4+ T lymphocyte count <200×10*6 cells/L [11]. In their study, compared to patients with CVID, patients with LOCID had a statistically higher prevalence of consanguineous families (8% vs. 29%), splenomegaly (31% vs. 64%), gastrointestinal disease (42% vs. 75%), granuloma (11% vs. 43%), and lymphoma (4% vs. 29%). The incidence of autoimmune cytopenia was 18%. Patients with LOCID required frequent antibiotic administration and hospitalization. Lymphocyte counts were lower, with a marked decrease in CD4+ cell counts and a significant decrease in the B cell compartment [11]. Our patient had lymphocytopenia, mild splenomegaly, and pneumonia. This could be a point of suspicion for a LOCID.

Our patient was initially suspected to have CVID due to hypogammaglobulinemia before treatment. Autoimmune cytopenia is the most common form of autoimmune complication. Most patients develop autoimmune cytopenia before or concurrent with the diagnosis of CVID. The management of autoimmune cytopenia in patients with CVID presents specific challenges, as the use of immunosuppressive agents can be contentious in that situation. Therefore, serum immunoglobulin testing is recommended for all patients with autoimmune cytopenia [18]. The mechanisms underlying autoimmunity in patients with CVID are heterogeneous. Dysregulated B cells are a hallmark of CVID. In addition, impaired T cell homeostasis has been reported to underlie the pathogenesis of one-third of CVID patients with autoimmune manifestations [18]. It is unknown whether these mechanisms are similar in patients with LOCID. LOCID may be under-recognized, as measurement of CD4 counts outside of HIV and transplant treatment is very rare, and there is a need for a shared consensus on the circumstances under which to test CD4+ count, as in our patient.

To the best of our knowledge, this is the first study to report the evaluation of a gene panel of PID using NGS in patients with LOCID. In CVID, monogenic defects have been identified in 10-50%. These monogenic defects are more likely to occur in patients with autoimmune complications [18]. It is also unknown whether these defects are shared between CVID and LOCID. We hope that research on the pathogenesis of LOCID will progress in the future.

A limitation of our case is that we did not assess CD4+ cell counts prior to ES treatment. However, we believe that the persistence of CD4+ deficiency and pre-treatment hypogammaglobulinemia over a period of 56 months after the end of treatment proves that it is not due to immunosuppressive therapy but to the original pathology. Owing to the diagnosis of LOCID, we found a need to prevent PCP, even after the end of treatment. This indicates the importance of evaluating the CD4+ count in some patients with AIHA/ES.

## Conclusions

In this case, severe CD4 lymphocytopenia with hypogammaglobulinemia was observed throughout the patient's course, even after treatment was completed. The possibility of AIHA/ES associated with immunodeficiency should always be considered. It is well known that we should check immunoglobulin class quantification to exclude CVID as an immunodeficiency causing AIHA/ES. However, in clinical practice, CD4+ cell count is not routinely checked in patients except in cases of HIV infection or transplantation. Had there been no awareness of LOCID, our case would have been recognized as CIVD and the need for PCP prophylaxis would have gone unnoticed. It may be necessary to consider the presence of CD4 dysfunction in patients with AIHA/ES, especially in those with low gammaglobulinemia, low pretreatment lymphocytopenia, concomitant infections, family history, and comorbidities such as splenomegaly. We hope that the pathophysiology, including genetic abnormalities, of LOCID will be better understood in the future to determine which cases require CD4 count evaluation.
